# Book Reviews

**Published:** 1892-12

**Authors:** 


### *BOOK REVIEWS.*

A Practical Treatise on Diseases of the Skin. By John V. Shoemaker, A. M., M. D., Professor of Skin and Venereal Diseases, Medicó Chirurgical College and Hospital of Philadelphia; Physician to the Philadelphia Hospital for Diseases of the Skin, etc., etc. Second Edition revised and enlarged, with Chromogravure plates and other illustrations. New York, D. Appleton & Co., 1892.

While Dr. Shoemaker has not the reputation of being purely a dermatologist his contributions, especially to the therapeutical literature of dermatology, have attracted considerable attention.

This work contains, first, a sufficiently complete description of the anatomy of the skin, this being followed by a section on physiology of the skin, and then general remarks upon symptomatology, diagnosis, pathology, ethology and treatment. The majority of the illustrations of the anatomical structure of the skin are below the average.

Under "pathology" the author goes quite thoroughly into bacteriology, quoting voluminously from authors who seem to have proven the specific action of certain micro-organisms, and also the opinions of others not so certain. He is not willing to express his opinion upon some of these views, contenting himself with giving both sides.

In "treatment" the author shows a leaning towards "constitutional," but not as specific, rather to the general improvement of the patient's condition. He reports good results, in proper cases, of the hypodermic injection of arsenic (sodium arseniate) following Radcliffe and Lipp.

There is a section on various baths, medicated and non-medicated; and a description of a hot air bath, a kind of a "sweat-box." Then comes a description of various medicated soaps. He believes lard, suet, lanolin (from sheep's wool) the best ointment bases, and quotes Liebreich and himself to show the advantages of the lanolin. He condemns the vaselines. Dr. Shoemaker also refers to the "oleates," preparations which he has gone to much trouble to evolve, in fact he deserves the credit for much good work in that direction.

There is the usual description of the use of and action of various drugs under "treatment." Massage, under a special heading, is a new section for skin works, and so, also, are the paragraphs upon various instrumental methods of treatment, and electricity.

He adheres to a "classification" of skin diseases. This common practice has, very properly, been condemned by some writer; it renders the study of dermatology more difficult, incurs the risk of finding itself wrong after later investigation, and does not afford any practical help to students, besides being often purely arbitrary.

The exanthemata, though more in the domain of general practice, are described. This is very well, if only for purposes of diagnosis. Chancroid, usually omitted, is, properly, described, it being a skin affection. Syphilis, as usual, is thoroughly gone into; the cuts of cases would not afford evidence for a diagnosis, however. Under the treatment he describes the method and form of mercurial treatment hypodermically, and also quotes results of introduction of mercury by electrical cataphoresis. He very properly describes rosacea without the too common "acne" prefix. Seborrhoic eczema, of Unna, is described under "Eczema." The theory of the sweat glands producing fat, and being the offending parts in the disease, is given. Lichen planus he places under "exudations," lichen ruber under hypertrophies. He substitutes "alopecia circumscripta" for "alopecia areata." The author quotes reports upon the use of Koch's lymph tuberculin, in lupus and allied diseases, but does not express his own opinion, as seems his custom. At the end of the work are two formularies, the first internal prescriptions, the last external, with the diseases in alphabetical order, each followed by prescriptions recommended for them.

To summarize briefly: The work shows quite extensive reading by the author, numerous authorities being quoted. You are often left to take your choice of the views held. Plates of skin diseases are practically useless. The author is a therapeutist, and "believes in drugs." Including the formulary there are over eight hundred pages in the work. Altogether, it would be a very useful addition to a physician's library. M. B. H.

REGIONAL ANATOMY IN ITS RELATION TO MEDICINE AND SURGERY. By George McClellan, M. D., Lecturer on Descriptive and Regional Anatomy at the Pennsylvania School of Anatomy; Professor of Anatomy in the Pennsylvania School of Fine Arts; Member of the Association of American Anatomists, etc. Illustrated by colored photographs of the author's own dissections. Vol. II. J. B. Lippincott Company, Philadelphia.

The second volume of Dr. McClellan's work fully sustains the high excellence of the first. The regions of the abdomen, perineum, back, pelvis, groin, hip, thigh, knee and popliteal space, leg, ankle and foot are herein described in that same happy manner which characterized the text of the first volume. All through this work we believe the reader will be struck with this feature —the simple and pleasing style in which the author describes what is ordinarily regarded as a dry subject. This probably has been Dr. McClellan's aim, and this may explain why in places not quite enough attention is paid to details. There is some nomenclature which differs from that of our standard text book (Gray), and which may be commended. The external and internal abdominal rings are called, respectively, the superficial and deep openings. The tensor *vaginæ* femoris is called (by preference) the tensor *fasciæ*. This name conveys to the beginner a better idea of the function of the muscle, besides creating no suggestion of any relation with another part which may be not very distant. We think the description of Hunter's canal, on page 255, might be improved upon. It is not quite right to say that Hunter's canal "ends in a rounded cord which is attached to the adductor tubercle of the femur." There seems to be some confusion here of the canal with the adductor magnus muscle and tendon. The author makes the quadriceps femoris muscle to be inserted into the patella, and then describes the ligamentum patellæ as the anterior ligament of the knee joint. A simpler teaching, and one well sustained by the action of the parts, is to regard the ligament as the tendon of the quadriceps (or better, triceps), with the patella, as a sesamoid bone developed in it. The plates in this volume, as a rule, are better than those in the preceding, those of the abdomen being especially distinct and accurate.

Dr. McClellan's work has been prepared during several years of patient and tedious labor, and he will be rewarded, we think, by seeing his book promptly take a high position among standard treatises on anatomy.

The mechanical execution by the Lippincott Company can hardly be improved upon.

---

The Essentials of Histology, Descriptive and Practical. For the use of Students. By E. A. Schäfer, F. R. S., Jodvell Professor of Physiology in University College, London. Third edition revised and enlarged. Over 300 illustrations. Philadelphia, Lea Bros. & Co. 1892.

This is one of the best, simplest and most practical manuals of histology extant. Beginning with the structure of the animal cells, the author gives instruction as to their form and elements, then the use of the microscope, the examination of common objects, which may confuse the student just beginning to examine specimens. The study of the blood corpuscles, their reaction to various agents; the blood corpuscles of amphibia also being considered. Then the various kinds of epithelium are studied, as are the connective tissues. Muscle, brain, nerves are described; blood vessels come next. The various glands, the skin, the heart, trachea and lungs and, in fact, every element and structure of the body is described.

The illustrations are clear and practical, the text gives a condensed description of the various structures without excessive verbiage. The work closes with a description of the methods used in preparing sections, denoting the agent best for hardening each tissue or organ, for section, then imbedding, cutting, staining (and how to make the stains) are given; finally how to mount the specimens for examination.

The reviewer has used the preceding edition of this work for some years, and has found none he likes so well. M. B. H.

---

Angel's Visits. By James W. Price, M. D., Atlanta, Ga. Foote & Davies, Atlanta, Ga.

This little volume by Dr. Price, which is dedicated to the medical profession, is a defence and elaboration of the idea that there does often exist in one way or another a communication between man on earth and angels in heaven. Milton said: "Millions of spiritual creatures walk the earth unseen." The work before us incorrectly accredits this quotation to Hesiod. The book is well written and a very entertaining discussion of this interesting matter. In addition to the above the volume also contains short essays of a pathetic and religious character, called "Whisperings from the Spirit World." Those who enjoy literature of this character will find the above a very acceptable contribution to the subject.

---

International Clinics. A Quarterly of Clinical Lectures, Etc. January, 1892. Philadelphia, J. B. Lippincott Co.

The International Clinics of the Lippincott Company have earned an established position in current medical literature. The present volume well sustains the promise of the earlier ones, containing valuable clinical lectures on subjects in which the active physician is almost daily interested. The lectures on the treatment of acute pneumonia, osteotomies for knock-knee and bow-legs, ataxia and destructive operations upon the fœtus are especially worthy of a careful reading. This issue also contains a memorial sketch of the late Dr. Charles T. Parkes, of Chicago.

---

Diseases of the Lungs, Heart and Kidneys. By N. S. Davis, Jr., A. M., M. D., Professor of Principles and Practice of Medicine, Chicago Medical College; Physician to Mercy Hospital; Member of the American Medical Association, Fellow of the American Academy of Medicine; Chicago Academy of Sciences, author of "Consumption, How to Prevent it and How to Live with it", etc. No. 14 *in the Physicians' and Students' Ready-Reference Series.* Philadelphia: The F. A. Davis Co., 1231 Filbert street.

This small volume is an elaboration of the notes used by Dr. Davis in his lectures on practice in the Chicago Medical College, and therefore it will have especial merit and usefulness for students. If anything more than the ability and reputation of the author were needed to commend the work, one has only to read the section on the diseases of the heart to recognize both the experienced physician and teacher. The diseases of the lungs, heart and kidneys are carefully and concisely described and especial attention is devoted to the treatment of the same.

